# A standardized approach for exact CT-based three-dimensional position analysis in the distal tibiofibular joint

**DOI:** 10.1186/s12880-021-00570-y

**Published:** 2021-03-06

**Authors:** Firas Souleiman, Martin Heilemann, Robert Hennings, Mareike Hennings, Alexis Klengel, Pierre Hepp, Georg Osterhoff, Annette B. Ahrberg

**Affiliations:** 1 Department of Orthopaedics, Trauma and Plastic Surgery, University Hospital Leipzig, Leipzig, Germany; 2 ZESBO - Centre for Research On Musculoskeletal Systems, University of Leipzig, Leipzig, Germany; 3 Department of Radiology, University Hospital Leipzig, Leipzig, Germany

**Keywords:** Syndesmosis, 3D Position control, Tibiofibular, Ankle, Clear space, CT

## Abstract

**Background:**

Assessment of tibiofibular reduction presents an intra- and postoperative challenge. Numerous two-dimensional measurement methods have been described, most of them highly dependent on leg orientation and rater. Aim of the present work was to develop a standardized and orientation-independent 3D based method for the assessment of syndesmotic joint position.

**Methods:**

In a retrospective single center study, 3D models of bilateral ankle joints, either after unilateral syndesmosis stabilization (operative group) or with no injury (native group) were superimposed (best fit matching) and aligned uniformly. Based on center of gravity calculations three orientation- and rater-independent parameters were determined: tibiofibular clears space (CS), vertical offset between both fibulae, and translation angle of the fibulae about tibia axis.

**Results:**

Bilateral CT datasets of 57 native and 47 postoperative patients were analyzed. In the native group mean CS was 2.7 (SD, 0.8; range, 0.7–4.9) mm, mean CS side difference was 0.62 (SD, 0.45) mm and mean translation angle was 1.6 (SD, 1.4) degrees regarding absolute values. The operative group was found to show a significantly higher CS side difference of 0.88 (SD, 0.75) mm compared to native group (*P* = *.046*). Compared to the healthy contralateral side, operated fibulae showed mean proximal displacement of 0.56 (SD, 1.67) mm (*P* = *.025*), dorsal displacement of 1.5 (SD 4.1) degrees (*P* = *.017*).

**Conclusion:**

By using 3D best fit matching, orientation- and rater-dependent errors can be minimized. Large interindividual and small intraindividual differences of uninjured couples support previous recommendations for bilateral imaging.

*Trial registration*: AZ 131/18-ek; AZ 361/19-ek

**Level of evidence:**

Level III.

## Background

Frequently, ankle fractures are associated with an injury to the distal syndesmosis in up to 45% of the cases [1–4]. Correct reduction of the fibula into the fibular notch of the tibia is a key factor for positive long-term outcome and a major evidence-based criterion of prognostic relevance in ankle fractures [5–11]. A lateral displacement of the fibula by 1 mm already reduces the contact area between talus and tibia by up to 40% [12–15]. Conventional radiography is unreliable to rule out malpositioning of the fibula. Post- or intraoperative bilateral computed tomography (CT) is the most sensitive and specific method to detect malreduction of the tibio-fibular joint [8–10, 16–26]. Even with CT, several different measurements are required to optimally assess the syndesmosis position [10, 22, 27–40]. Schon et al. described three measurements for rotation, lateral and anterior-posterior orientation of the fibula as most specific CT measurements of ankle syndesmotic malreduction [41]. The results of their study presented surprisingly low native side-to-side correlation across all 35 tested measurement methods. Overall, the methods varied considerably in their ability to detect malreduction and an accurate reduction. Proper positioning of the fibula is essential in order to restore the anatomic relationships [42].

Since three-dimensional (3D) assessment has become increasingly important in surgery in recent years, the aim of this study was to establish a standardized 3D based measurement technique for evaluation of the syndesmosis and to provide reference values for this procedure. Furthermore, this methodology should be independent on leg orientation differences and rater independent regarding landmark detection. The major hypothesis of this study was that side differences were significantly higher in unilateral surgically treated ankles compared to native ankles in terms of 3D parameters developed. Secondary, necessity of bilateral CT for exact position control of the syndesmosis should be assessed.

## Methods

The study protocol has been approved by the local institutional review board (Ethical Committee at the Medical Faculty, Leipzig University, AZ 131/18-ek; AZ 361/19-ek). In a retrospective single center (Level I Trauma Center) study, 47 consecutives bilateral CT examinations of patients with a unilateral syndesmotic injury (operative group) in the period from 2008 to 2017 and 57 bilateral CT examinations of uninjured ankle joints (native group) in the period from 2010 to 2019 were included.

Demographic data of interest were age, gender and injured side. In the operative group, all CT examinations were performed after ankle fixation with a syndesmotic screw (3.2 mm, DePuy-Synthes, West Chester, PA), or a suture-button device (TightRope®, Arthrex, Naples, FL). Inclusion criteria of the operative group were AO 44 B and C fractures with intraoperative pathological hook test and stabilization of the syndesmosis, anatomic fixation of the fracture components, an inconspicuous contralateral side and imaging of at least 10 cm of the distal lower leg as well as the entire talus. Exclusion criteria were missing postoperative bilateral CT, trauma of the non-operated side in the past, insufficient CT scans that did not include the fixation device.

Scan types for the native group were CT angiographies with intravenous contrast medium of either the lower extremities (n = 52) or the whole body including the lower extremities (n = 5). Patients in both groups were older than 18 years and had no relevant bony pathology besides the fracture in the operative group.

CT imaging was performed with two different multidetector CT scanners (operative group: iCT 256; native group: Ingenuity 128; Philips, Best, The Netherlands). Routine scan parameters varied between the scanners and scan types: Tube current of 100 mAs to 226 mAs, tube voltage of 100 kV or 120 kV, collimation of 64 mm × 0.625 mm or 128 mm × 0.625 mm, pitch of 0.400 to 0.759 and a rotation time of 0.750 s to 0.925 s. In-plane resolution was < 1 mm. Slice thickness of axial reformations was 0.67 mm to 2 mm. Only CT examinations that show the bony structures at least 10 cm proximal to the distal tibia plateau were evaluated.

All patients had given informed consent before surgery and CT scan. In the operative group, open reduction and fixation of the ankle fractures was performed through a lateral approach to the fibula with a lag screw and neutralization plate. In case of additive medial malleolar fractures, a standard medial approach with open reduction and screws, plate or both have been performed.

In case of a posterior tibial fragment (Volkmann’s triangle), nine patients were operatively stabilized by a dorsal plate or indirect by anterior–posterior lag screws. Syndesmotic instability was tested by the hook test under fluoroscopy [43–45]. In case of instability the surgeon chose between a transsyndesmotic screw and a suture-button device (TightRope®) by preference. Standard fluoroscopy (lateral and mortise view) was applied intraoperatively to identify the reduction.

### Assessment of the 3D models

After the operative treatment CT data sets of native and operative group were segmented using a 3D image processing software (Mimics 22.0, Materialise, Leuven, Belgium). In case of enclosed implants, these were virtually removed. Resulting holes were filled virtually with respect to the anatomical geometry. Finally, stereolithographic models (STLs) were created and exported for three-dimensional measurement.

### Data transformation and parameter determination

Data transformation and parameter determination were executed by using Geomagic Design X 2016 (3D Systems, Rock Hill, SC). Prior to any parameter determination, a standardized global alignment of the lower leg bones had to be established. In this context, two planes orthogonal to the scanning axis, the first 50 mm proximal and the second 100 mm proximal to the tibial articular surface of the left leg, were regarded. The centroids of the tibial cross sections within both planes were calculated and the straight line connecting both points defined the left tibia axis, representing the z-axis for each dataset, respectively. This axis definition prevents parameter discrepancies due to different global orientation of different datasets.

Following this, both STLs, tibia and fibula, were merged for both sides. Hence, the relative position of the fibula with respect to the tibia of the same side was fixed. The right side was mirrored and superimposed with the contralateral side by best fit matching. The distal 100 mm of both tibiae served as relevant geometry for the matching step. An area of this size was used to ensure accurate matching results even in case of extensive tibial injuries. This procedure enabled to visually identify misalignments between both fibulae.

**Fig. 1:**
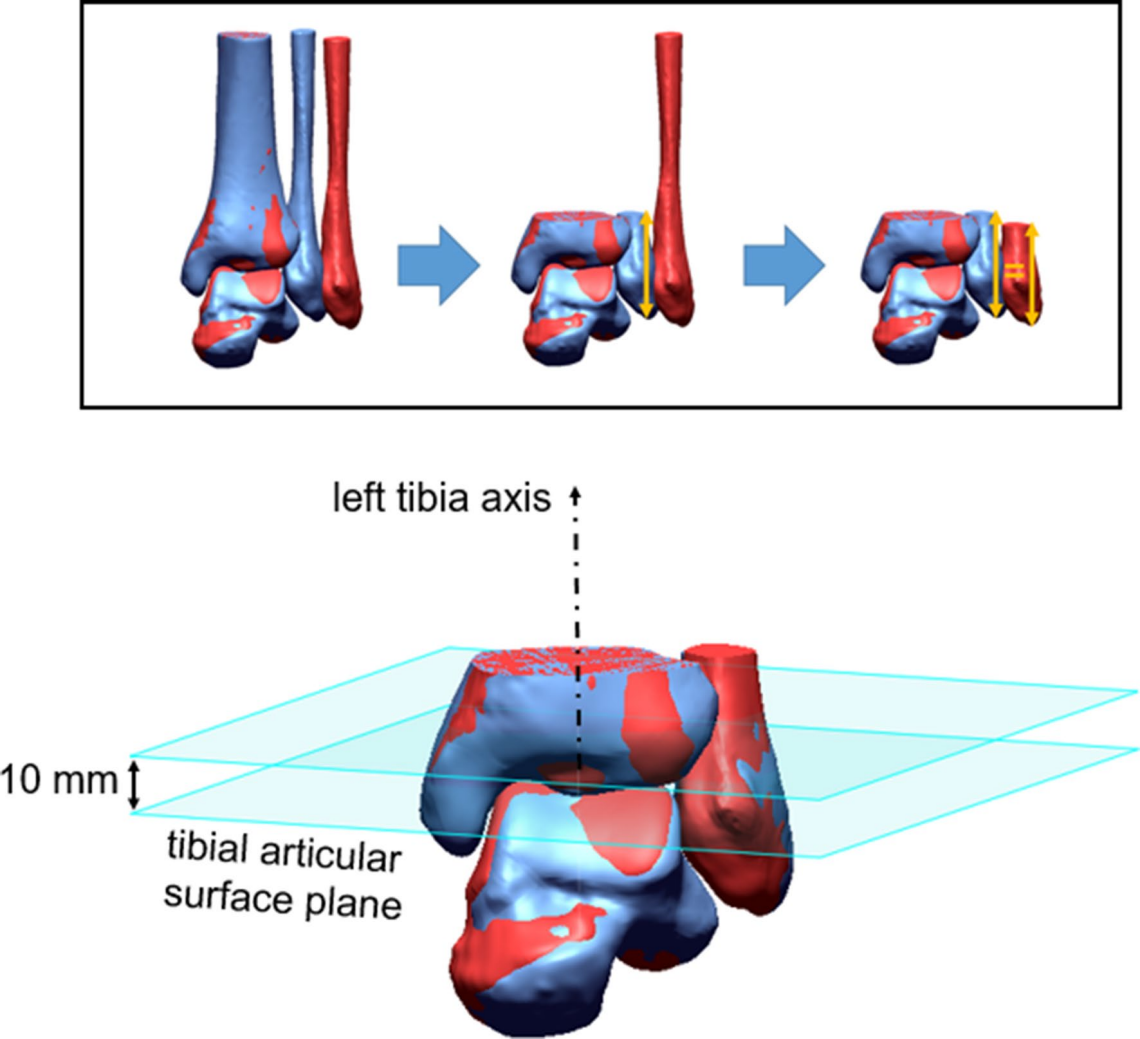
Best fit matching and subsequent cutting of both sides. Right fibula is excessively shifted in favor of visibility in the upper illustration. Resulting cutted 3D data including left tibia axis and corresponding planes is shown in the lower illustration

Since the distal syndesmosis was the area of interest, left tibia, left fibula, and right tibia were cut 20 mm proximal to the tibial articular surface of the left leg. With regard to center of volume calculations, to be implemented in the following, the right fibular part had to be as long as the left one. Differences in length between both fibulae would have a considerable effect on center of volume calculations and, thus, disturb actual measurements. Therefore, the length of the left fibula was measured and then the right fibula was cut at same length (Fig. 1). By contrast, the distal tibial parts were assumed to be equally long, since superimposition of both sides due to best fit matching was performed based on tibial geometry. STLs were transformed to solids by methods of reverse engineering.

**Fig. 2:**
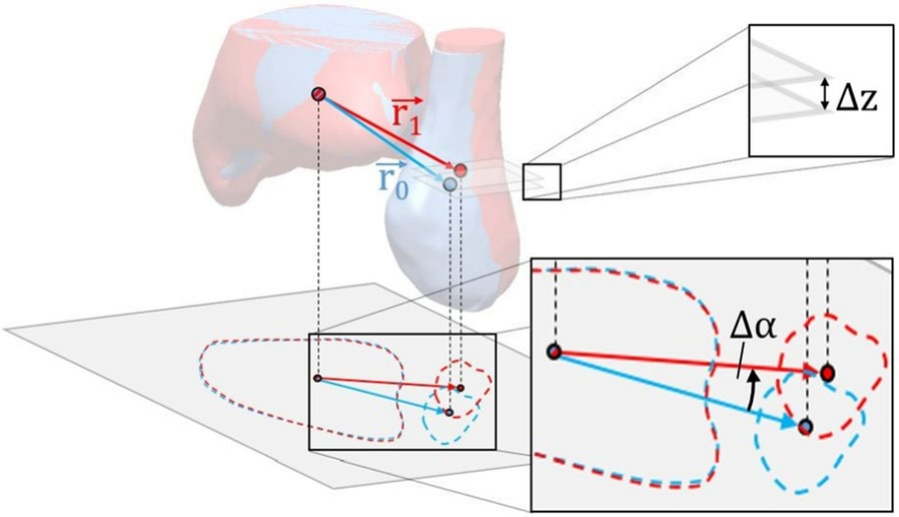
Visualization of tibial and fibular centers of volume, respective connection vectors, and measurement of vertical offset (Δz) and translation angle (Δα)

Subsequently, centers of volume of tibial and fibular part were calculated for both sides. The left side was set as reference for the native group, the uninjured side for the operative group. Connection vectors of tibial and fibular centers of volume on reference side ($\overrightarrow{{r}_{0}}$) and contralateral side ($\overrightarrow{{r}_{1}}$) were used to identify three-dimensional misalignments between both fibulae relative to superimposed tibia alignment. This three-dimensional misalignment was quantified by two parameters: vertical offset ($\Delta z$) along previously defined left tibia axis and translation angle ($\Delta \alpha$) about this axis (Fig. 2).

Since left tibia axis represents the z-axis of the coordinate system, fibular offset is equal to the z-component of the difference vector of both center of volume connections:

$$\Delta z={r}_{1}^{(z)}-{r}_{0}^{(z)}$$

Positive differences imply proximal offset with respect to the reference fibula, negative differences imply distal offset.

Fibular rotation about tibia axis is defined as the angle between the projection of both connection vectors into a perpendicular plane, for example the plane defined by $z=0$, and can be calculated via cross product of both vectors:

$$\Delta \alpha =\mathrm{arcsin}\left(\frac{{r}_{0}^{(x)}\cdot {r}_{1}^{\left(y\right)}-{r}_{0}^{(y)}\cdot {r}_{1}^{(x)}}{\sqrt{{{r}_{0}^{(x)}}^{2}+{{r}_{0}^{(y)}}^{2}}\cdot \sqrt{{{r}_{1}^{(x)}}^{2}+{{r}_{1}^{(y)}}^{2}}}\right)$$

Positive angles imply external rotation about tibia axis, negative angles imply internal rotation.

**Fig. 3:**
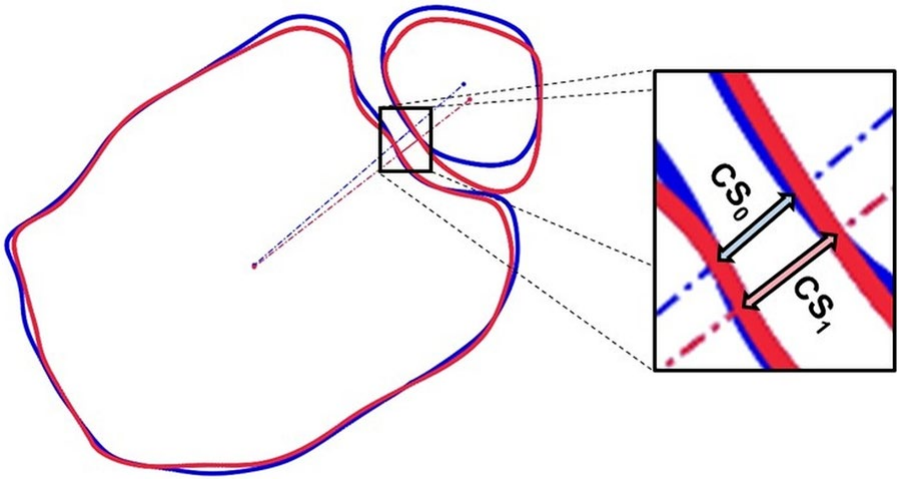
Visualization of tibiofibular clear space measurement along the connection line of tibial and fibular cross section centroids for reference and contralateral side

Furthermore, a landmark independent measurement of tibiofibular clear space was implemented. Clear space was regarded within the plane, perpendicular to left tibia axis, 10 mm proximal to the tibial articular surface of the left leg. Tibial and fibular centroids within this plane were calculated. The straight line through tibial and fibular centroids for both sides intersect tibial and fibular bone contour. Distance between these two intersections defines the clear space in our study (Fig. 3). Tibiofibular clear space difference ($$\Delta CS$$) is defined as difference between reference side ($${CS}_{0}$$) and opposite side ($${CS}_{1})$$:

$$\Delta CS={CS}_{1}-{CS}_{0}$$

### Statistical analysis

Statistical data analysis was performed by using Matlab (Mathworks, Natick, MA) and G*Power (HHU Düsseldorf, Germany). Absolute values of all parameters were compared regarding native and operative group. Wilcoxon rank-sum tests (one-tailed) were used to check statistical significance of group-specific differences. Furthermore, operative parameters were compared to 0 mm or 0°, respectively, in order to identify some systematical operative misalignments: oversized / undersized clear space, proximal / distal vertical offset, external / internal translation angle. Those directions were indicated by the sign of the parameters. One-sample *t *tests (two-tailed) were used to check statistical significance of those directions. Level of significance was set at *P* < *0.05* for both test types.

## Results

Demographic data of the native and operative patient groups are shown in Table 1. Age and gender distribution of both groups were comparable. According to the AO classification, there were 19 AO 44 B and 28 AO 44 C fractures in the operative group [46].

**Table 1:** Demographic data of all patients

Group	Number	Gender		Age in years		
		Male	Female	Mean	SD	Min | Max
native	57	39	18	53	18	18 | 90
operative	47	30	17	48	18	24 | 87
*P* value		.68			.19	

The 114 legs of the native group showed a mean tibiofibular clear space of 2.7 (SD, 0.8) mm. Measured native clear spaces ranged from 0.7 mm to 4.9 mm. All 47 operatively treated legs showed a mean tibiofibular clear space of 3.5 (SD, 1.7; range, 0.1–9.1) mm, whereas contralateral uninjured leg showed a mean clear space of 3.2 (SD, 1.0; range, 1.6–5.8) mm.

In order to evaluate tibiofibular reduction, clear space differences between both legs were analyzed. The median tibiofibular clear space difference, regarding absolute values, was 0.55 mm for the native group and 0.68 mm for the operative group.

**Fig. 4:**
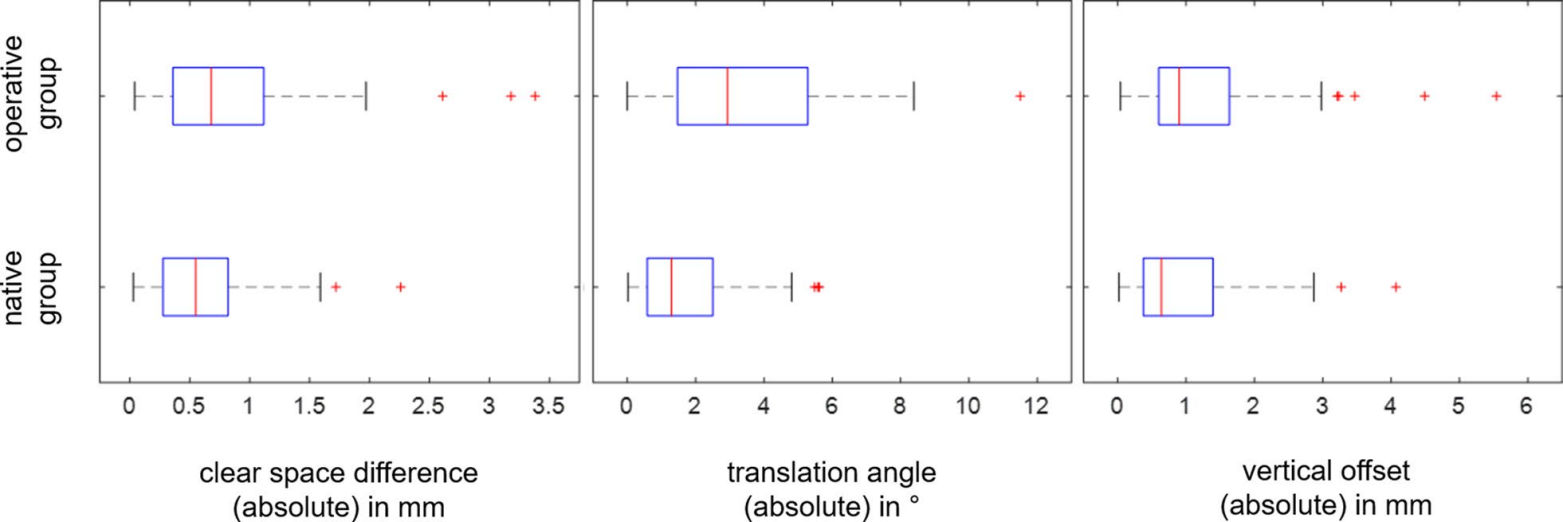
Boxplots regarding absolute values of tibiofibular clear space difference, vertical offset and translation angle for native and operative group

Furthermore, vertical offset and translation angle with respect to the reference side were used to identify misalignments. Boxplots in Fig. 4 show absolute values of clear space difference, vertical offset and translation angle for native and operative group, respectively. In the operative group, a clear space difference exceeding 2 mm was found in 3 of 47 patients (6.3%), a vertical offset exceeding 3 mm in 5 patients (10.6%), and a translation angle exceeding 5 degrees in 12 patients (25.5%). In the native group, a clear space difference exceeding 2 mm was found in 1 of 57 patients (1.7%), a vertical offset exceeding 3 mm in 2 patients (3.5%), and a translation angle exceeding 5 degrees in 3 patients (5.2%).

**Fig. 5:**
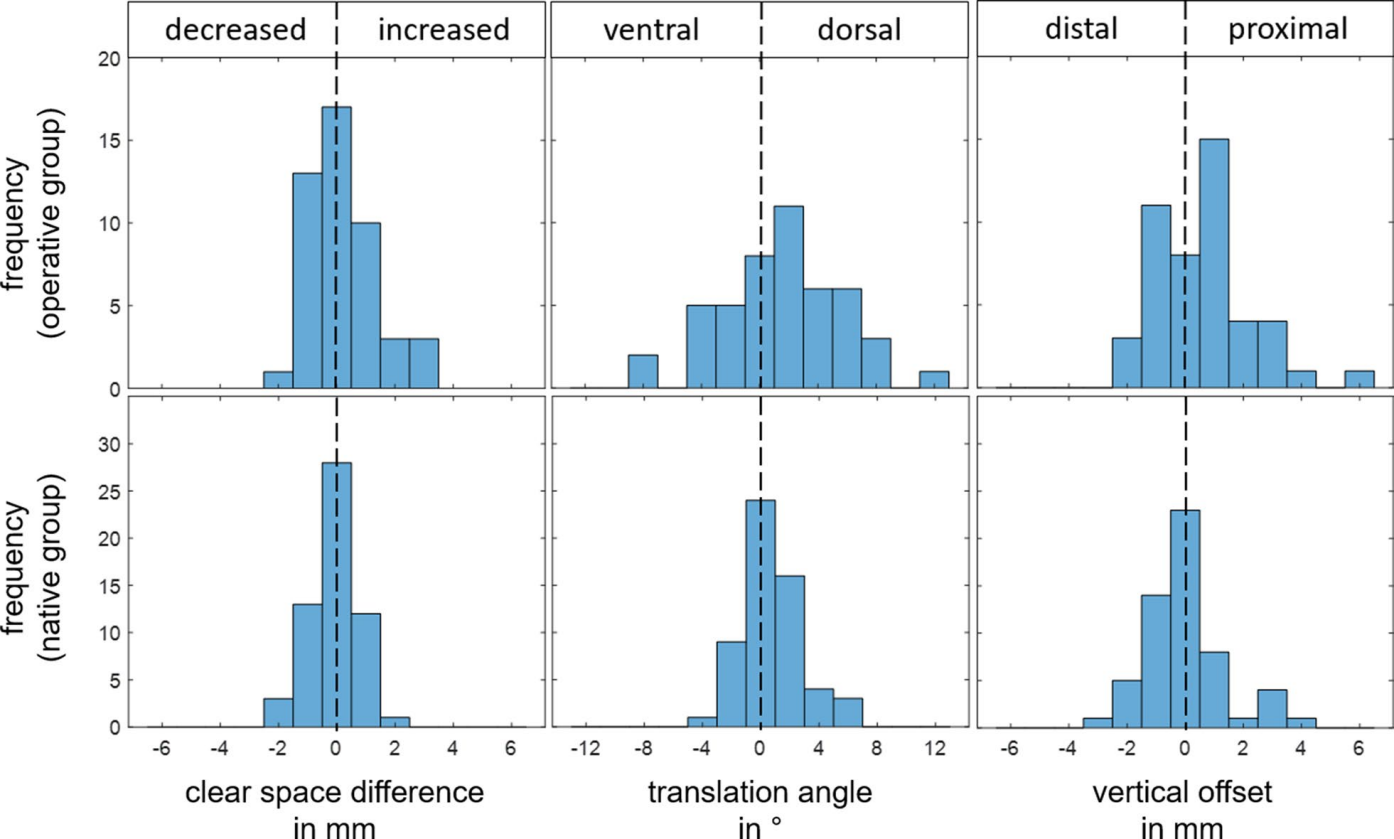
Histograms regarding tibiofibular clear space difference, vertical offset and translation angle for native and operative group with consideration of their direction

Figure 5 shows histograms for all three parameters with consideration of their sign. This allows identification of systematic misalignment direction of operative group in comparison to the native group.

Hypothesis tests and decisions are shown in Table 2. According to those tests, absolute tibiofibular clear space difference, vertical offset, and translation angle of operative group are significantly larger compared to native group. Furthermore, directions of misalignment after surgery regarding vertical offset and translation angle were identified. Mean vertical offset is +0.56 (SD, 1.67) mm and significantly different from zero which indicates proximal offset. Mean translation angle is +1.5 (SD, 4.1) degrees and also significantly different from zero which indicates external rotation. Mean tibiofibular clear space difference after surgery is +0.23 (SD, 1.14) mm, but with the numbers available, no significant direction of misalignment could be detected. A post-hoc power analysis showed a power of 0.27 for this hypothesis test.

**Table 2:** Hypothesis tests ($\Delta CS$: tibiofibular clear space difference, $\Delta z$: vertical offset, $\Delta \alpha$: translation angle, n: native group, o: operative group)

H_0_	H_1_	Test	*P* value	decision
$\left|{\Delta CS}_{o}\right|=\left|{\Delta CS}_{n}\right|$	$\left|\Delta {CS}_{o}\right|>\left|{\Delta CS}_{n}\right|$	Wilcoxon rank-sum test	.046	H_0_ rejected
$\left|{\Delta z}_{o}\right|=\left|{\Delta z}_{n}\right|$	$\left|{\Delta z}_{o}\right|>\left|{\Delta z}_{n}\right|$	Wilcoxon rank-sum test	.029	H_0_ rejected
$\left|{\Delta \alpha }_{o}\right|=\Delta \left|{\alpha }_{n}\right|$	$\left|{\Delta \alpha }_{o}\right|>\left|{\Delta \alpha }_{n}\right|$	Wilcoxon rank-sum test	< .001	H_0_ rejected
${\Delta CS}_{o}=0$	${\Delta CS}_{o}\ne 0$	One-sample *t* test	.167	H_0_ accepted
${\Delta z}_{o}=0$	${\Delta z}_{o}\ne 0$	One-sample *t* test	.025	H_0_ rejected
${\Delta \alpha }_{o}=0$	${\Delta \alpha }_{o}\ne 0$	One-sample *t* test	.017	H_0_ rejected

## Discussion

The main objective of the work, the development of a three-dimensional measurement method for the exact assessment of the syndesmosis position in order to eliminate limitations of the previously used methods, was achieved. Since all three developed parameters showed significantly larger side differences in the operative group compared to the native group, the major hypothesis of this study was confirmed.

Slightly different orientation of both legs of a patient is an issue in two-dimensional imaging processes, and thus resulting image planes are tilted against each other. This results in an error in 2D parameter calculations such as clear space measurement [47]. In the present study, these intraindividual differences in leg orientation were compensated by using three-dimensional datasets, mirroring one side and subsequent best fit matching of both legs. Subsequent considerations of particular planes do not include the tilting error described above.

In addition, three-dimensional data enable a more comprehensive characterization of differences in position compared to two-dimensional data.

Still, interindividual differences in orientation remained an issue. Therefore, one reference tibia axis was determined for each dataset using centroid calculation of tibial cross sections in two clearly defined planes orthogonal to the scanning axis. Although these two planes are subject to potentially slight leg orientation differences, the calculation of the tibial cross-sectional centroids in these planes is almost uninfluenced due to the uniform geometry of the tibia in those regions. Even in a slightly tilted cross-sectional plane, the center of gravity remains centrally located within the tibial geometry. Hence, this procedure enabled consistent axial alignment of each dataset on the basis of respective tibia geometries. Thus, substantially increased interindividual comparability was achieved.

In previous measurement methods anatomical landmarks were selected by an examiner, which resulted in rater dependent measurement differences [10, 22, 27–40]. In contrast, the methods described in this study avoid rater dependent landmark detection. Instead, centers of volume and area were calculated based on respective three-dimensional geometries and two-dimensional cross sections. Centers of volume were used to calculate the rater independent parameters vertical offset and translation angle. An additional substantial advantage of the centers of volume assessment is its stability to local artifacts of the geometry in contrast to landmark based measurements. Centroids of tibial cross sections enabled rater independent measurement of tibiofibular clear space. Clear space, after three-dimensional adaptation, was used as one of the most frequently used parameters to describe tibiofibular reduction. In order to also describe the remaining two dimensions, the parameters vertical offset and translation angle were introduced. In consequence, displacement in all three dimensions were described.

The translation angle is mainly determined by displacement of the fibula in ventral or dorsal direction. This angle is actually also influenced by possible medialization / lateralization. However, for small angles such as those in the study, this influence is negligible. In addition, medial / lateral displacement is quantified using the clear space differences. Height displacements of both fibulae are exactly described by the vertical offset.

Applying 3D based measurements on unilaterally stabilized ankle joints, a noticeably wider distribution was found compared to the native group. In addition, hypothesis tests confirmed significantly increased absolute values of all three parameters for the operative group. Moreover, absolute results for the native group indicated thresholds for all three parameters according to usual outlier definition using 150% of the interquartile range plus the third quartile. Rounded to the nearest integer, following thresholds resulted: 2 mm for clear space difference, 3 mm for vertical offset and 5 degrees for the translation angle. This is comparable to other clinical and biomechanical studies, where side differences larger than 2 mm are considered relevant [12, 26, 28, 48, 49].

For the operative group, 20 exceedances of those parameter threshold values were detected.

Consideration of the sign of the measured parameters additionally allowed identification of direction of misalignments compared to reference side. According to this study the fibula of the treated leg was significantly proximally shifted and dorsally inclined about tibia axis. This observed systematic direction of malalignment should be considered during intraoperative reduction. Whether this influences the outcome of the surgery remains to be investigated. With regard to differences in tibiofibular clear space, no general direction was determined for treated legs. However, the test power for this hypothesis was fairly low (0.27) due to the small effect size.

There is also a controversial discussion about the necessity of bilateral CT control after syndesmosis stabilization [9, 10, 21, 24, 26, 28]. Regarding clear spaces measured in this study, the native group exhibited a standard deviation of 0.84 mm whereas the median clear space difference between both sides was 0.55 mm. This indicates a substantially larger interindividual clear space variation compared to intraindividual variation, and thus supports the recommendation from the literature for bilateral imaging.

The authors acknowledge some limitations to the design of the study. The presence of implants that were virtually removed and injured bone structure may affect the accuracy of the CT measurements. CT angiographies were used for the native group, since numerous bilaterally uninjured data sets were available. However, the measurement error resulting from scanning parameter variations was acceptably low in both groups [50]. Furthermore, the consideration of intraindividual side differences also ensures that the calculated parameters are barely influenced by the respective scan type. Other assessment methods including plain radiography, weight-bearing CT/MRI and dynamic testing were not evaluated in this study. It is conceivable that the effects on the measurement parameters in weight-bearing CT/MRI could be greater.

No outcome scores were collected during the study. Thus, no clinical symptoms of complaints can be deduced from the measurement results.

In summary, established measuring methods [41] for assessing syndesmosis can be transferred to a three-dimensional measurement. By using 3D models, best fit matching and center of volume/centroids calculations, leg orientation dependent errors and rater dependent errors can be minimized while all aspects of syndesmosis malreduction will be described sufficient. This is a crucial advantage over two-dimensional measuring methods [42]. Hence, the presented methodology provides diverse potential in future use cases. It can be applied as a diagnostic tool to determine preoperative and postoperative displacement of injured ankle that may be suitable for surgery. It can be used for preoperative planning of correction. The described three-dimensional measurement method, automated on intraoperative 3D imaging, could also be used in the future as a tool for accurate position analysis in real time, thus reducing the number of revision surgeries and providing additional safety to the surgeon. Further measurements are necessary to create a comprehensive three-dimensional database.

## Data Availability

The datasets used and/or analyzed during the current study are available from the corresponding author on reasonable request.
